# Gemcitabine fixed-dose rate infusion for the treatment of pancreatic carcinoma: a meta-analysis of randomized controlled trials

**DOI:** 10.1186/s13000-014-0214-8

**Published:** 2014-11-25

**Authors:** Jiqing Xie, Jing Yuan, Laichun Lu

**Affiliations:** Department of Pharmacy, General Hospital of Jinan Military Command, Jinan, 250031 China; Department of Medical Information, General Hospital of Jinan Military Command, Jinan, 250031 China; Department of Pharmacy, Institute of Surgery Research, Daping Hospital, Third Military Medical University, Changjiang Road 10, Chongqing, 400042 China

**Keywords:** Gemcitabine, Fixed-dose rate, Pancreatic adenocarcinoma, Meta-analysis

## Abstract

**Background:**

Pre-clinical evidence shows that fixed dose rate (FDR) infusion of gemcitabine could optimize plasma concentration of gemcitabine, while the clinical efficacy and toxicity of FDR infusion of gemcitabine in advanced pancreatic carcinoma has not been systematically investigated. Thus, this meta-analysis was designed to ascertain this issue.

**Methods:**

Databases of EMBASE, PubMed, and Cochrane Library were searched for eligible randomized controlled trials (RCTs). RCTs comparing FDR and standard 30-min infusion of gemcitabine in advanced pancreatic carcinoma were included. The primary endpoints were treatment efficacy (overall response rate, 1-year survival rate, median survival, and time to treatment failure) and toxicities were secondary endpoints (neutropenia, thrombocytopenia, anemia, and vomiting). Relative risks or mean differences and corresponding 95% confidence intervals (CIs) were calculated.

**Result:**

After careful and rigorous selection, 3 eligible RCTs including 764 patients of advanced pancreatic adenocarcinoma were included in this meta-analysis. For treatment efficacy, FDR gemcitabine provided significantly longer median survival over standard gemcitabine (Mean Difference = 1.24 months, 95% CI: 0.39-2.09), while there was no statistical difference in other endpoints of treatment efficacy. For toxicities, patients with FDR gemcitabine experienced significantly more grade 3/4 hematological toxicities than those received standard gemcitabine (neutropenia, thrombocytopenia, and anemia), while there was no difference for vomiting.

**Conclusion:**

Compared with standard 30-min infusion, FDR gemcitabine provide longer median survival, but increased the risk of hematological toxicities for patients with advanced pancreatic adenocarcinoma.

**Virtual Slides:**

The virtual slide(s) for this article can be found here: http://www.diagnosticpathology.diagnomx.eu/vs/13000_2014_214

**Electronic supplementary material:**

The online version of this article (doi:10.1186/s13000-014-0214-8) contains supplementary material, which is available to authorized users.

## Background

Pancreatic adenocarcinoma is one of the most aggressive solid malignancies and the eighth most common cause of cancer death worldwide [1]. Due to early metastatic dissemination, the prognosis of pancreatic adenocarcinoma is extremely poor and the 5-year survival rate is only 6% [2].

The majority of pancreatic adenocarcinoma patients are diagnosed at advanced stage and when diagnosed, the tumor is often metastasized. Therefore, systemic chemotherapy is often the treatment of choice. The administration of gemcitabine is widely used due to the evidence of clinical benefit and prolonged survival over fluorouracil [3]. While the objective response rate and survival of gemcitabine chemotherapy is still disappointing. Gemcitabine is usually administered in a manner of 30 minutes intravenous infusion, while pharmacokinetic evidence suggests that fixed-dose rate infusion (10 mg/m^2^/min) of gemcitabine could optimize plasma concentration of gemcitabine [4–6]. In 2012, Qiu et al. reported FDR gemcitabine could not improve survival or response rate over standard gemcitabine in non-small cell lung cancer [7]. For pancreatic adenocarcinoma, FDR gemcitabine and standard gemcitabine have been compared in several randomized clinical trials (RCTs) [8–10], but no meta-analysis has been performed to systematically investigate the effects of FDR gemcitabine on treatment efficacy or toxicity. Thus, this meta-analysis was designed to compare the difference between FDR gemcitabine and standard gemcitabine in the treatment efficacy and toxic effects among patients with advanced pancreatic adenocarcinoma.

## Methods

### Searching strategy

Electronic databases of MEDLINE (via PubMed), EMBASE (via Ovid), and Cochrane Library were searched and the latest search was performed on September 2014. The searching strategy consisted of the following medical subheadings and key words: “pancreatic neoplasms”, “pancreas cancer”, “pancreatic cancer”, “fixed-dose rate”, “fixed dose rate”, “fixed-dose-rate”, “fixed dose-rate”, “prolonged infusion” and “prolonged constant infusion”. The detailed searching strategy was shown in the Additional file 1. References of eligible trials and relative reviews were also manually searched. There was no limitation in databases searching.

### Inclusion criteria

Trials met the following criteria were included: (a) randomized controlled trials; (b) patients were pathologically confirmed of pancreatic carcinoma at advanced stage; (c) comparing FDR infusion (10 mg/m2/min) and standard infusion (30 min) of gemcitabine; and (d) published with full-text articles. Two investigators (Jiqing Xie and Jing Yuan) selected eligible trials met above criteria independently. Disagreement between two investigators was solved by discussion until consensus was achieved.

### Data abstraction

Two investigators (Jiqing Xie and Jing Yuan) extracted data from eligible trials independently with a pre-designed data extraction form. And disagreement was discussed with another expert (Laichun Lu) until consensus was achieved. The following data were abstracted from each eligible clinical trial: first author, publishing time, country, chemotherapy regimens, number of patients, age, number of male, number of patients with metastatic pancreatic carcinoma, number of patients with complete response and partial response, 1-year survival rate, median survival, time to treatment failure, grade 3/4 hematological and non-hematological toxicities. Tumor response was measured according to Response Evaluation Criteria in Solid Tumors (RECIST). National Cancer Institute Common Toxicity Criteria version 3.0 was used for toxicity reporting. The research protocol was in strict compliance with the institutional guidelines of the Ethics Committee at the Third Military Medical University.

### Validity assessment

Methodology quality of included trials was also assessed using the “risk of bias” tool recommended by Cochrane Handbook V5.0.2. Sequence generation, allocation concealment, blinding, incomplete data and selective reporting were assessed. Based on the methods reported in each trial, each of above five components was graded “yes”, “unknown” or ”no”, which meant low risk of bias, uncertain of bias and low risk of bias respectively.

### Statistical analysis

The primary endpoints were treatment efficacy (overall response rate, 1-year survival rate, median survival, and time to treatment failure) and toxicities were secondary endpoints (neutropenia, thrombocytopenia, anemia, and vomiting). Mean differences were calculated for median survival and time to treatment failure. Relative risks (RRs) were calculated for the other endpoints. 95% confidence intervals (95% CI) were also calculated for all endpoints. A RR with a 95% CI without 1 and the mean difference with a 95% CI without 0 is considered of statistical significance. Fixed-effects model was used for calculating RRs and median differences unless heterogeneity was significant across trials [11]. A RR >1 indicates that FDR infusion is associated with a higher incidence of response, 1-year survival rate and lower incidence of grade 3/4 toxicities; and a mean difference >0 suggests that the FDR infusion of gemcitabine increases the median survival and time to treatment failure. Heterogeneity was tested using the Q-statistic and I^2^. A P value less than 0.1 or I^2^ > 50% indicated that there existed significant heterogeneity [11]. A funnel plot was used to test publication bias [12,13]. All statistical analyses were carried out by Review Manager 5 (Version 5.2, The Cochrane Collaboration).

## Results

**Figure 1:**
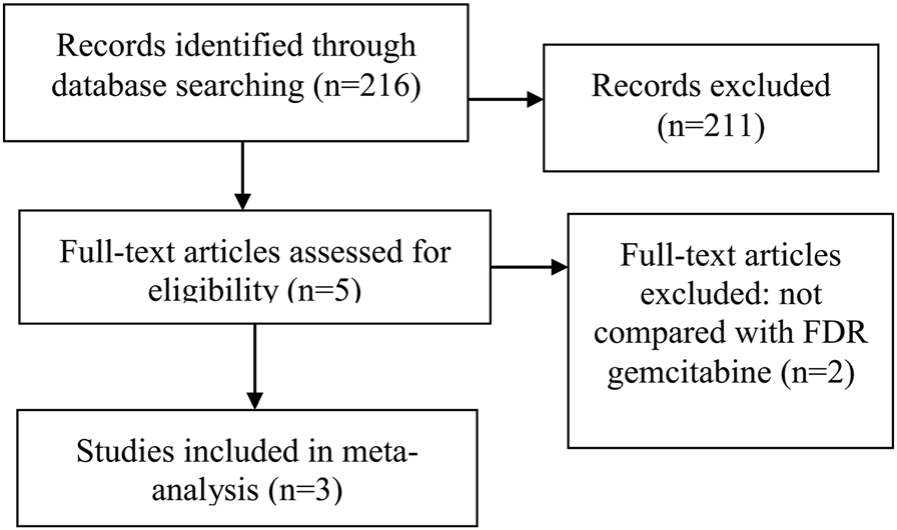
**Flow Diagram of Study Selection.**

After comprehensive database searching, 216 records were retrieved. By screening titles and abstracts and further reviewing of full-text papers, 3 eligible RCTs were included and analyzed in this meta-analysis. The process of study selection was shown in Figure 1. The baseline characteristics of eligible trials were shown in Table 1. 764 patients with confirmed pancreatic adenocarcinoma contributed to the pooled meta-analysis. Most of enrolled patients had metastatic pancreatic carcinoma.

**Table 1:** **Baseline characteristics of eligible trials**

**Author**	**Year**	**Country**	**Num** ^**a**^	**Male**	**Age** ^**b**^	**Metastasis**	**Chemotherapy**
**Kulke MH**	2009	USA	58	38	58.9(31–81)	58	gemcitabine 1500 mg/m^2^ infusion 10 mg/m^2^/min on days 1, 8, and 15, every 28 days
			62	35	58.9(36–84)	62	gemcitabine 1000 mg/m^2^ 30-minute infusion d1, 8, and 15;Cisplatin 50 mg/m^2^ 30-minutes infusion on days 1 and 15, every 28 days
**Poplin E**	2009	USA	277	160	61(36–87)	246	gemcitabine 1500 mg/m^2^ infusion at 10 mg/m^2^/min days 1, 8, and 15 every 28 days cycle
			275	155	64(31–88)	248	gemcitabine 1000 mg/m^2^/30 min weekly for 7 week, rest 1 week, then 1000 mg/m^2^/30 min weekly for 3 week and rest 1 week
**Tempero M**	2003	USA	43	27	62(42–85)	38	gemcitabine 1500 mg/m^2^ infusion at a rate of 10 mg/m^2^/min, d1, 8, and 15
			49	32	62(31–89)	46	gemcitabine 2200 mg/m^2^ infusion over 30 min,d1, 8, and 15

a: Number of patients analyzed, b: data are present as median and range.

### Primary endpoints

**Figure 2:**
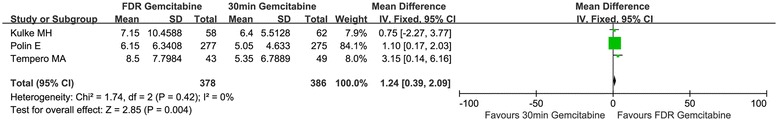
**Forest plot for the comparison of median survival.** FDR gemcitabine provided longer median survival than standard gemcitabine (Mean Difference = 1.24 months, 95% CI: 0.39-2.09). FDR: fixed-dose rate; SD: standard deviation; CI: confidence intervals.

The primary object of this meta-analysis was to compare the differences of treatment efficacy between FDR gemcitabine and standard gemcitabine. To this end, we calculated the pooled 1-year survival rate, median survival, and overall response rate. As shown in Table 2, FDR gemcitabine provided a significantly long median survival (Mean Difference = 1.24 months, 95% CI: 0.39-2.09 months; Figure 2) than standard gemcitabine, while there was no significant difference in overall response rate, 1-year survival rate, or time to treatment failure. As shown, no significant heterogeneity between studies were detected. Visual assessment of funnel plot did not reveal any evidence of publication bias (Figures not shown).

**Table 2:** **Meta-analysis results**

**Outcome**	**RR** ^**a**^	**Heterogeneity**
**Primary Endpoints**		
Overall response rate	1.65 [0.99, 2.75]	P = 0.45, I2 = 0%
1-year survival rate	1.35 [0.98, 1.86]	P = 0.12,I2 = 59%
Median Survival	1.24 [0.39, 2.09]^b^	P = 0.42,I2 = 0%
Time to Treatment Failure	0.54 [0.25, 1.33]^b^	P = 0.22,I2 = 33%
**Secondary Endpoints**		
Neutropenia	1.79 [1.49, 2.15]	P = 0.92,I2 = 0%
Thrombocytopenia	2.19 [1.64, 2.93]	P = 0.50,I2 = 0%
Anemia	1.68 [1.15, 2.45]	P = 0.45,I2 = 0%
Vomiting	1.31 [0.89, 1.93]	P = 0.79,I2 = 0%

a: RR with a 95% CI without 1 and the mean difference with a 95% CI without 0 is considered of statistical significance; b: for median survival and time to treatment failure, mean differences were calcluated.

### Secondary endpoints

**Figure 3:**
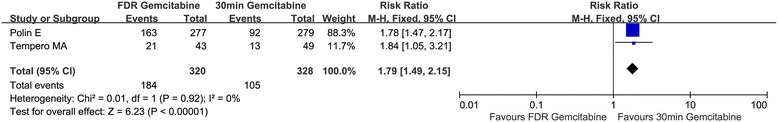
**Forest plot for the comparison of neutropenia.** FDR gemcitabine significantly increased the risk of neutropenia (RR = 1.79, 95% CI: 1.49-2.15). FDR: fixed-dose rate; CI: confidence intervals.

Grade 3/4 toxic events were the secondary endpoints of this meta-analysis. Neutropenia, thrombocytopenia, anemia and vomiting were analyzed. As shown in Table 2, compared with standard gemcitabine, FDR gemcitabine significantly increased the incidence of hematologic toxicities (neutropenia, thrombocytopenia, and anemia; compassion of neutropenia was shown in Figure 3), while there was no difference in the incidence of vomiting. As shown in Table 2, no evidence of heterogeneity was found. Funnel plots also showed no publication bias (Figures not shown).

## Discussion

In this meta-analysis, we found FDR gemcitabine, compared with standard gemcitabine, could provide longer median survival for patients with advanced pancreatic carcinoma, and patients received FDR gemcitabine would experience more hematological toxicities.

Burris HA and colleagues reported in 1997 that single-agent gemcitabine administered as weekly 30-min infusion could offer significantly longer survival and clinical benefit than 5-fluorouracil [3]. After that gemcitabine has been the standard chemotherapy for advancer pancreatic carcinoma. Gemcitabine (2’,2’-difluorodeoxycytidine) is a prodrug and potent cytotoxic agent. The deoxycytidine kinase phosphorylate gemcitabine to gemcitabine monophosphate, and subsequent phosphorylation steps yielded gemcitabine diphosphate and gemcitabine triphosphate, which are active metabolites of gemcitabine [4,14]. The gemcitabine triphosphate could inhibited cell proliferation and induce apoptosis by incorporation into DNA [15,16]. The deoxycytidine kinase is the rate-limiting enzyme in the accumulation of the active diphosphate and triphosphate metabolites [4]. Grunewald et al. and Abbruzzese et al. demonstrated that the dose rate about 10 mg/m2/min could maximize the rate of formation of gemcitabine triphosphate for mononuclear cells [4,6]. In addition to these pharmacokinetic studies and preclinical studies, Tempero et al. reported a randomized phase II trial in 2003 and found FDR gemcitabine significantly increased 1-year and 2-year survival rates compared with 30-min gemcitabine [10]. In 2009, Poplin and colleagues showed that FDR gemcitabine could not provide substantially improved survival or symptom benefit over 30-min gemcitabine [9]. Thus, we conducted this meta-analysis to compare the treatment efficacy and toxicity between FDR gemcitabine and 30-min gemcitabine in patients with advanced pancreatic adenocarcinoma.

In this meta-analysis, we found FDR gemcitabine could only increase median survival of patients with pancreatic adenocarcinoma over standard gemcitabine, and there was no difference in overall response rate, 1-year survival rate, or time to treatment failure. This could be explained by the following reasons. First, the sample size or the number of eligible trials of this meta-analysis was small. As shown in Table 2, the results of overall response rate (RR = 1.65, 95% CI: 0.99-2.75) and 1-year survival rate (RR = 1.35, 95% CI: 0.98-1.86) were marginally significant, and it was highly possible that the results of response and 1-year survival would be statistically significant when further trials are reported. Second, most patients enrolled in this meta-analysis had metastatic disease (Table 1) and the prognosis of metastatic pancreatic cancer is very poor. This could explain why the median survival was significantly longer for patients received FDR gemcitabine but the 1-year survival rate was not significantly different. Because the prognosis for metastatistic patients was extremely poor and most patients died with one year. For toxicities, patients with FDR gemcitabine experienced substantially higher incidence of hematologic toxicities (neutropenia, thrombocytopenia, and anemia) than those with 30-min gemcitabine, but there was no difference in the incidence of vomiting. Compared with 30-min gemcitabine, the infusion time was prolonged for FDR gemcitabine (120 min vs. 30 min), and the exposure time for blood cells was longer. Thus, the results for hematologic toxicities were expectable. Additionally, it has also been demonstrated that FDR gemcitabine could increase the incidence of hematologic toxicities in non-small cell lung cancer by Qiu et al. [7].

This is the first meta-analysis comparing treatment efficacy and toxicities between FDR gemcitabine and standard 30-min gemcitabine in pancreatic adenocarcinoma. No significant heterogeneity was found and funnel plots revealed no evidence of publication bias. Additionally, the evidence level of this meta-analysis was evaluated by the GRADE profiler, which facilitates the clinical application of our results. On the other hand, the major limitation of this meta-analysis lies in that the sample size of this meta-analysis was small. To include all potential trials, we designed comprehensive searching strategy and searched the 3 comprehensive databases. In spite of the effort to perform a comprehensive meta-analysis, only 3 eligible trials were included. Further studies are warranted to validate our findings.

## Conclusions

In conclusion, our meta-analysis demonstrated that compared with standard 30-min gemcitabine, FDR gemcitabine provided longer median survival but there was no significant difference in overall response rate and 1-year survival rate in patients with advanced pancreatic adenocarcinoma, while FDR infusion was associated with more grade 3/4 hematological toxicities.

## Additional file

Additional file 1:
**Searching Strategy of this meta-analysis.**
