# Supplementary material for: Gemcitabine fixed-dose rate infusion for the treatment of pancreatic carcinoma: a meta-analysis of randomized controlled trials
Source: Diagn Pathol. 2014 Nov 25;9:214. doi: 10.1186/s13000-014-0214-8 (PMC4251942; doi:10.1186/s13000-014-0214-8)
Supplement: Additional file 1: — Searching Strategy of this meta-analysis. [file 13000_2014_214_MOESM1_ESM.docx]

**Searching Strategy**

(((randomized controlled trial[pt]) OR (controlled clinical trial[pt]) OR (randomized[tiab]) OR (placebo[tiab]) OR (randomly[tiab]) OR (trial[tiab])) NOT (animals[mh] NOT humans[mh])) AND ((pancreatic neoplasms) or (pancreas neoplasms) or (pancreas cancers) or (pancreatic cancer) or (pancreatic carcinoma)) AND (gemcitabine) AND ((fixed-dose rate) OR (fixed dose rate) OR (fixed-dose-rate) OR (fixed dose-rate) OR (prolonged infusion) OR (prolonged constant infusion))
